# Structural Features of Alkaline Extracted Polysaccharide from the Seeds of *Plantago asiatica* L. and Its Rheological Properties

**DOI:** 10.3390/molecules21091181

**Published:** 2016-09-06

**Authors:** Jun-Yi Yin, Hai-Hong Chen, Hui-Xia Lin, Ming-Yong Xie, Shao-Ping Nie

**Affiliations:** 1 State Key Laboratory of Food Science and Technology, Nanchang University, Nanchang 330047, China; junyi86@163.com (J.-Y.Y.); chh0810ncu@163.com (H.-H.C.); myxie@ncu.edu.cn (M.-Y.X.); 2 Xiamen Huaxia College, Xiamen 361024, China; lin1986410@163.com

**Keywords:** seeds of *Plantago asiatica* L., alkaline extracted polysaccharide, structure, rheology

## Abstract

Polysaccharide from the seeds of *Plantago asiatica* L. has many bioactivities, but few papers report on the structural and rheological characteristics of the alkaline extract. The alkaline extracted polysaccharide was prepared from seeds of *P.* *asiatica* L. and named herein as alkaline extracted polysaccharide from seeds of *P. asiatica* L. (PLAP). Its structural and rheological properties were characterized by monosaccharide composition, methylation, GC-MS and rheometry. PLAP, as an acidic arabinoxylan, was mainly composed of 1,2,4-linked Xyl*p* and 1,3,4-linked Xyl*p* residues. PLAP solution showed pseudoplastic behavior, and weak gelling properties at high concentration. Sodium and especially calcium ions played a significant role in increasing the apparent viscosity and gel strength.

## 1. Introduction

Arabinoxylan is widely distributed around the world. It is non-starch polysaccharide, and composed of a linear β-1,4-linked Xyl*p* backbone. Ara residues are usually distributed in the side chain. Residues of β-1,4-linked Xyl*p* in the backbone are substituted at some *O*-2 and/or *O*-3 by Ara and other residues. Arabinoxylan is usually considered to play in beneficial roles in stimulating prebiotic growth [1,2], reducing cardiovascular risk [3], and exhibiting immunoregulation [4] and anti-tumor [5] activities. Psyllium, a mucilaginous polysaccharide material from seed husks, is also an excellent source of arabinoxylan [6,7,8,9,10].

There are more than 200 species of *Plantago*, which are used extensively all over the world. Polysaccharide extracted from *Plantago* seeds is one of bioactive components which were widely reported in recent years [11]. Previous studies mainly focused on the polysaccharide extracted from *Plantago* genus, to characterize its rheological behavior and gelling properties [12,13,14]. However, as yet few reports has paid enough attention to the polysaccharide extracted from *P. asiatica* L., except for a very limited number of reports in which the water-soluble polysaccharide has been confirmed as a typical arabinoxylan [15,16] that exhibited weak gelling property [17] and promoted colon health [18,19,20].

Therefore, the aim of this paper was to investigate the structural and rheological properties of alkaline extracted polysaccharide. The alkaline extracted polysaccharide (named hereafter as PLAP) was prepared from the seeds of *P.* *asiatica* L. Techniques including monosaccharide compositional analysis, methylation analysis, together with gas chromatography-mass spectrometry (GC-MS), were combined to characterize the molecular structural features of the PALP. Additionally, the rheological properties of PALP with an addition of different ratio of sodium or calcium ions were also characterized by rheometry. Relationships between the structural features and rheological performance of the *P.* *asiatica* L. polysaccharide are discussed.

## 2. Results

### 2.1. Structural Characterization

Table 1 shows the basic molecular structural features, in which PALP was characterized to be acidic polysaccharide that contained 20.5% of uronic acid. Xyl and Ara, whose molar ratio was 4.1, were considered as the main compositional monosaccharides since they accounted for a significantly higher proportion of the contents. In contrast, Rha, Glc and Ga detected at low levels were the minor compositional monosaccharides for the PALP. These results indicated that PALP should be considered an arabinoxylan. In addition, the intrinsic viscosity of PALP diffused in 0.1 M NaCl solution was measured at 5.81 dL/g. The molecular weight of PLAP would be much higher than 3.80 × 10^−6^ as the sample recovery was only 13.9% when it was subjected for HPSEC analysis. The low sample recovery could be explained by the phenomenon that the polysaccharide hardly passed through the filter membrane. Most of the sample was retained by the filter.

The glycosyl-linkage composition results of PLAP are presented in Table 2. The main residues in PLAP were T-linked Ara*f* (5.06%), 1,3-linked Ara*f* (10.22%), T-linked Xyl*p* (10.88%), 1,3-linked Xyl*p* (11.39%), 1,4-linked Xyl*p* (6.19%), 1,2,4-linked Xyl*p* (13.30%) and 1,3,4-linked Xyl*p* (38.98%). 1,2,4-linked Xyl*p* and 1,3,4-linked Xyl*p* were the primary branched sugar residues, indicating side chains probably connected to the backbone through *O*-2 and *O*-3 positions of 1,4-linked Xyl*p* residues. PLAP might be highly branched, since it was found to be rich in 1,2,4-linked Xyl*p* and 1,3,4-linked Xyl*p* residues. Other residues, such as T-linked Gal*p*, 1,4-linked Gal*p*, 1,2-linked Rha*p* and 1,3-linked Glc*p*, were also detected in small amounts. According to previous reports [12,13,14], this kind of polysaccharide from *Plantago* seeds should be considered as an arabinoxylan whose backbone was probably composed of 1,4-linked Xyl*p*, including 1,2,4-linked Xyl*p* and 1,3,4-linked Xyl*p*.

### 2.2. Rheological Properties of PLAP

#### 2.2.1. Steady State Shear Properties

**Figure 1:**
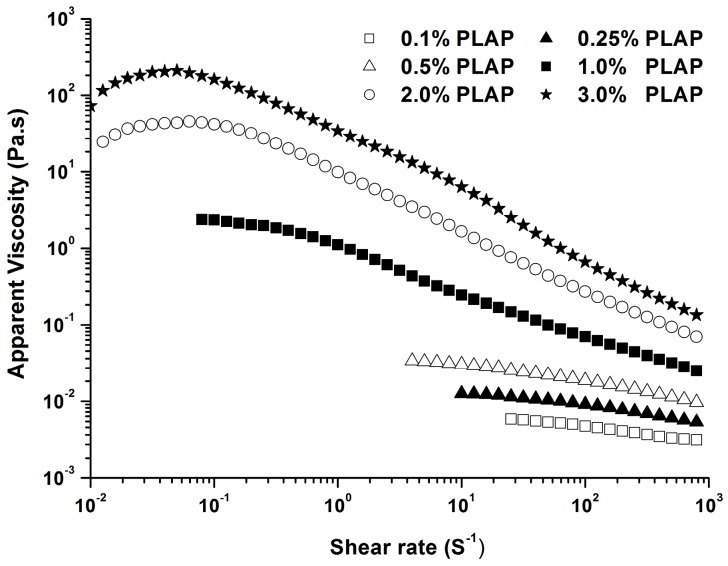
Effect of polysaccharide concentration on apparent viscosity of PLAP at 25 °C.

The flow curves for a series of concentrations of PLAP ranging from 0.1% to 3.0% in aqueous solution at 25 °C are shown in Figure 1. The typical curves of PLAP solutions showed shear thinning behavior, as the viscosity decreased with increasing shear rate. It was more obvious when the concentration was higher than 1.0%. For lower concentrations, the viscosity depended less on shear rate. Similar effects of concentration on viscosity of arabinoxylan from *Plantago* family [12,13] and other resources [21,22] has also been observed.

**Figure 2:**
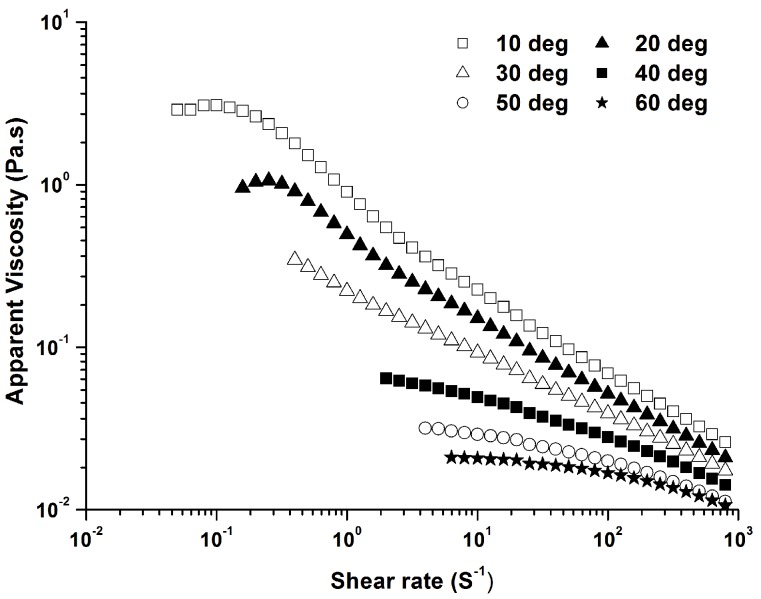
Effect of temperature on apparent viscosity of 1.0% PLAP.

The polysaccharide solutions showed pseudoplastic behaviour as the temperature increased, but its apparent viscosity decreased dramatically with the increasing temperature (Figure 2). Several interactions occur for polysaccharides in solution, such as hydrogen and electrostatic bonds, and entanglements. When the disruption rate of these interactions reaches a similar level as their generation rate, a constant apparent viscosity is observed, named the Newtonian plateau. A Newtonian region in the low shear rates was observed, especially at the temperature of 60.0 °C. Both the Cross and Carreau flow models could be used to describe the shear-thinning behavior of polymer solutions, and calculate zero shear rate viscosity (η_0_). Herein, the Cross flow model was used to evaluate the flow behavior of PLAP solutions at different concentrations.

The Cross equation is as follows:

(1) η=η∞+η0−η∞(1+αγ)m

where η is the apparent viscosity, η_0_ is the zero shear rate viscosity which is obtained by measuring the viscosity at a range of low shear rate and extrapolating to zero shear rate, η_∞_ is the limiting viscosity infinite shear rate, α is a time constant related to the relaxation time of polysaccharide in solution, *m* is a dimensionless exponent. The Cross equation generally describes well the shear rate dependence of aqueous polysaccharide solutions. It was chosen to calculate η_0_ and relaxation time. The values of the parameters obtained by fitting above equation to the tested data are shown in Table 3 and Table 4, respectively. An increase of η_0_ and α values with the increase of polysaccharide concentration was found, while both of them decreased as the temperature went up. The relaxation time (α value) decreased with increasing temperature, suggesting that formation of new interactions between molecules predominated over the rate of their disruption.

The Arrhenius equation is usually taken to describe the temperature dependence of the apparent viscosity for an ideal Newtonian liquid, as shown in the following equation:

log η = log A + E_a_/RT (2)

where η is the apparent viscosity, A is a constant, E_a_ is the activation energy for viscous flow, and R is the gas constant. According to this equation, E_a_ could be calculated at zero-shear rate viscosity which was estimated by the Cross equation (log η = 5220/T − 17.53, R² = 0.989). The value of E_a_ was calculated to be 43.40 kJ/mol.

#### 2.2.2. Viscoelastic Properties

**Figure 3:**
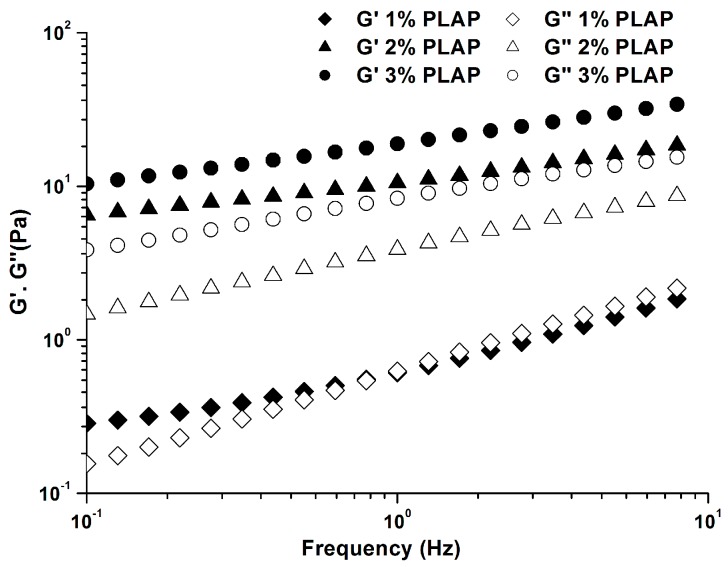
Effect of polysaccharide concentration on storage (G′) and loss (G″) moduli of PLAP at 25 °C.

The mechanical spectra (frequency dependence of storage modulus (G′), and loss modulus (G″)) of PLAP at 25 °C are shown in Figure 3. For the frequency sweep tests, the strain used in all the experiments was within the linear viscoelastic region where the gel structure was not damaged. PLAP showed typical weak-gel structure: the values of G′ were higher than those of G″ during the whole experimental range [23], except for 1.0% PLAP. Both G′ and G″ showed a dependency on frequency and concentration. The higher the concentration, the less dependency the G′ on frequency (Figure 3). The increase in G″ as a function of polysaccharide’s concentration suggested a higher viscosity contribution to the gel structure. More solid-like behavior was observed for those solutions at high concentrations. This may be due to an increased number of junction zones between the polymer chains at higher concentration [24,25]. A higher number of intramolecular junctions at lower concentration do not contribute to intermolecular cross-linking.

**Figure 4:**
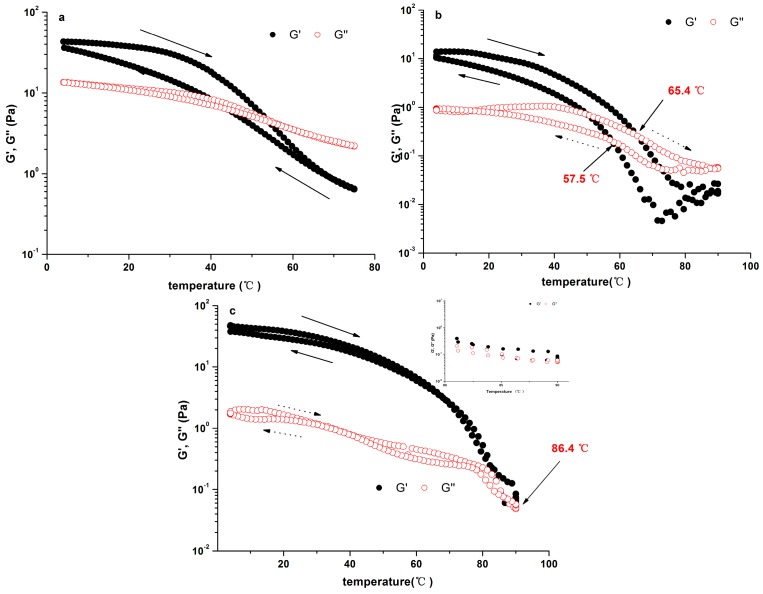
Variation in G′ and G″ of polysaccharide during heating and cooling by 2 °C/min. (**a**) without salt-3.0% PLAP; (**b**) with 0.2 M Na^+^-1.0% PLCP; (**c**) with 0.2 M Ca^2+^-1.0% PLCP.

Figure 4a presents the change of G′ and G″ of 3.0% PLAP as the temperature ranged from 5 to 78 °C. The heating and cooling curve (G′) of PLAP was not superimposed. As the temperatures increased during the range lower than 30 °C, both G′ and G″ of PLAP decreased gradually, then, both of them decreased sharply when the temperature was higher. There was a cross-over point for G′ and G″ at the temperature of 55 °C. The gel melting process was completed. There was thermal hysteresis demonstrated in the cooling curve of PLAP. It seemed that the gel structure was weak, unlike the gel fraction from psyllium reported by Guo et al. [13].

### 2.3. Effects of Na^+^ and Ca^2+^ on Rheological Properties of PLAP

**Figure 5:**
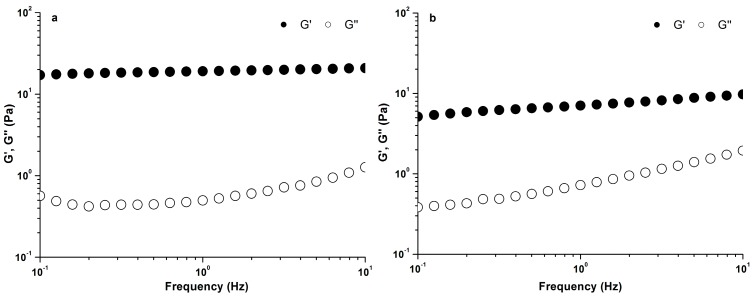
Mechanical spectra showing variation of G′ and G″ with frequency of 1.0% PLAP with 0.2 M Ca^2+^ (**a**) or 0.2 M Na^+^ (**b**).

The gelling properties of PLAP were studied when the polysaccharide solution was added with 0.2 M Na^+^ or Ca^2+^. The effects of Ca^2+^ and Na^+^ on the frequency dependence of G′ and G″ of PLAP are shown in Figure 5. It appeared that the strain dependence when the strain increased over 100%. Therefore, a strain of 5% was applied in the tests to make sure it was within the linear viscoelastic region. G′ was higher than G″, and showed a weak dependence on frequency throughout all the frequency range. The spectrum of G′ and G″ of PLAP with 0.2 M Ca^2+^ showed relatively less frequency dependence, suggesting PLAP with 0.2 M Ca^2+^ was more typical of a true gel.

**Figure 6:**
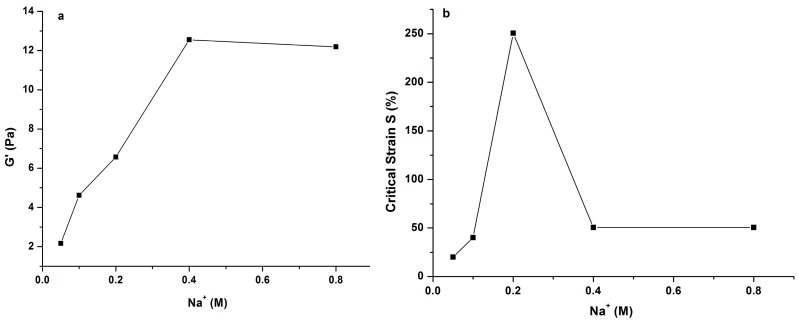
Variation of elastic modulus (G′) (**a**) and critical strain (S) (**b**) of 1.0% PLAP added with 0.2 M Na^+^.

**Figure 7:**
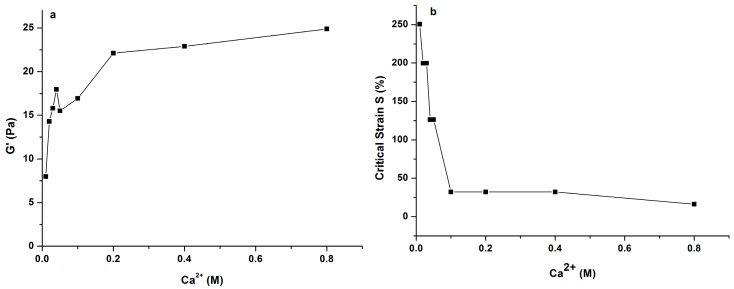
Variation of elastic modulus (G′) (**a**) and critical strain (S) (**b**) of 1.0% PLAP added with 0.2 M Ca^2+^.

In order to deeply understand the influence of Na^+^ and Ca^2+^ concentration on gelling properties of PLAP, elastic modulus G′ and critical strain (S) of the polysaccharide were compared by adding different concentrations of Na^+^ or Ca^2+^, using strain sweep tests. The critical strain S was defined as the strain where G′ decreased sharply in the test, reflecting the deformability of a gel, and G′ was obtained in the linear region of the gel. The results are shown in Figure 6 and Figure 7, respectively. It was found that G′ values increased greatly with increasing of Ca^2+^ concentration, especially when it was lower than 0.04 M. Then, the G′ value decreased when Ca^2+^ concentration was 0.05 M. The gel strength (G′) increased again with increasing concentration of Ca^2+^, but the rate was a little slower. As for the critical strain S, it obviously decreased with increasing Ca^2+^ concentration. When the concentration of Ca^2+^ was higher than 0.05 M, critical strain S was almost stable.

When it came to Na^+^, it was obviously different. The gel strength was much weaker than that of PLAP with Ca^2+^ at the same ion concentration. It increased slowly, and did not increase any more when the concentration of Na^+^ was higher than 0.4 M. For critical strain S, it reached the highest value when the concentration of Na^+^ was 0.2 M. Then, it decreased with increasing of Na^+^ concentration.

On the basis of the above results, it is indicated that the mixture of PLAP solution with 0.2 M Ca^2+^ was more typical of a true gel. Figure 4b,c show the changes of G′ and G″ with temperature for 1% PLAP addition with Na^+^ or Ca^2+^. At low temperature, both G′ and G″ of the polysaccharide solution changed gradually. When the temperature increased, G′ and G″ values of PLAP addition with 0.2 M Na^+^ decreased more obviously than that of PLAP containing 0.2 M Ca^+^. The heating and cooling curves of 1.0% PLAP added with 0.2 M Ca^2+^ were superimposed, different from the original solution or the polysaccharide solution added with 0.2 M Na^+^, where both of them exhibited pronounced thermal hysteresis phenomenon. A sharp decrease in G′ and G″ above ~50 °C for 1.0% PLAP addition with 0.2 M Na^+^ and ~70 °C for 1.0% PLAP addition with 0.2 M Ca^2+^ was observed. This phenomenon suggested characteristic gel melting. The melting process changed gradually, and the cross point was detected at 65.4 °C for the sample addition with 0.2 M Na^+^. It was 86.4 °C for 1.0% PLAP addition with 0.2 M Na^+^. These results suggested that the gel of 1.0% PLAP addition with Ca^2+^ was more stable during the temperature increasing.

## 3. Discussion

Polysaccharide extracted from *Plantago* seeds is usually arabinoxylan, although it was reported to contain small amount of uronic acid [7,8,15,16,26,27,28]. In this study, the alkaline extracted polysaccharide from the seeds of *P. asiatica* L. was confirmed to be highly branched arabinoxyaln and contain uronic acid, which was in accordance with polysaccharide from other psyllium resources. Intrinsic viscosity is a measure of the hydrodynamic volume occupied by the polysaccharide chains in a certain solvent. Few studies have reported intrinsic viscosity information of psyllium polysaccharide, except some data on arabinoxylan from maize, rye and wheat. The intrinsic viscosity of PLAP was 5.81 dL/mg in 0.1 M NaCl, which was close to that of the water-extracted fraction from the same sources [29]. It was also similar to values of some arabinoxylan reported by Rattan et al. [30] and Cui and Mazza [31], but slightly higher than other reported intrinsic viscosities that ranged from 2 to 4 dL/g [22,32]. The botanical differences, sample preparation, together with the uronic acid contents may combine to account for the different intrinsic viscosity.

A shear thinning liquid is a kind of non-Newtonian fluid as its apparent viscosity decreases with increasing shear rate. PLAP solutions at higher concentration showed more significant shear thinning behavior. The shear thinning appeared at lower shear rate, and the viscosity reduction is more pronounced for 3.0% PLAP than those with lower concentration. The shear thinning behavior was also widely reported for polysaccharides from other psylliums [12,13,14,17]. Although a strong hydrogen bonding exists among chains of β-1,4-linked xylose [31], the presence of arabinose side residues would reduce interaction among arabinoxylan chains which lead to less aggregation and lower viscosity of polysaccharide solution. This could explain why the highly branched PLAP only showed weak gel properties. However, both the apparent viscosity and gel strength of PLAP were much higher than that of arabinoxylan from cereals [21,22,30]. The high molecular weight of the polysaccharide may be one of the important reasons for this.

The shear viscosity and gel structure of polysaccharide solutions containing carboxylic acid groups are sensitive to ionic strength. Both water-extracted polysaccharide [17] and the alkaline-extracted fraction from the seeds of *P. asiatica* L. showed weak gel properties. An addition of Na^+^ or Ca^2+^ could significantly increase their gel strength. Some differences of rheological features, such as the divergence in apparent viscosity and gel strength, may be attributed to structural differences between the two fractions. Alkaline treatment may be another reason because de-esterification reactions probably occurred, which could break di- and triferulate crosslinks between polymer chains, and single ferulic acid residues [32]. As we know, gelation of some polymers is thermoreversible as gels could be formed during the cooling process of hot solutions and reversibly melted during heating process. The setting and melting temperature could be characterized as thermoreversible. When the melting temperature is higher than the setting temperature, the gel is considered to exhibit thermal hysteresis [33]. There was thermal hysteresis for 3.0% PLAP. Although both the melting and setting temperature of 1.0% PLAP with an addition of 0.2 M Na^+^ was higher than that of 3.0% PLAP, there was still thermal hysteresis. As for the gel of 1.0% PLAP added with 0.2 M Ca^2+^, the melting temperature was almost equal to its setting temperature. This means Ca^2+^ plays a more significant role than Na^+^ does in altering the thermal properties of PLAP gel.

## 4. Materials and Methods

### 4.1. Materials

The seeds of *P. asiatica* L. were purchased from Ji’an County (Ji’an, Jiangxi Province, China) and dried before use. Monosaccharide standards of mannose (Man), rhamnose (Rha), ribose (Rib), galactose (Gal), xylose (Xyl), arabinose (Ara), fucose (Fuc) and glucose (Glc) were obtained from Sigma Chemical Co. (St. Louis, MO, USA). Aqueous solutions were prepared with ultra-pure water from a Milli-Q water purification system (Millipore, Bedford, MA, USA). All other reagents were of analytical grade.

### 4.2. Polysaccharide Preparation

Seeds of *Plantago asiatica* L. were first extracted with boiling water twice to remove the water-extracted polysaccharide. Then, the residue was extracted twice with 0.5 M NaOH for 2 h at 4 °C. The combined extract was filtered, neutralized with acetic acid, and concentrated on a rotary evaporator at 55 °C. The concentrated solution was added ethanol at a final concentration of 80%. Then, the polysaccharide was redissolved in water, and deproteinated according to the Sevag method [34]. The resulting aqueous solution was dialyzed, and precipitated again with ethanol (80%, *v*/*v*). After centrifugation, the precipitate was successively washed with anhydrous ethanol, acetone and diethyl ether. Final obtained polysaccharide PLAP was subjected to vacuum drying.

### 4.3. Physicochemical Characteristics

PLAP were analyzed for sugar contents [35,36], uronic acid contents [37] and protein contents [38]. Intrinsic viscosity determination was performed at 25.0 °C in 0.1 M NaCl solvent, using an Ubbelohde Capillary Viscometer (Cannon Institution Company, State College, PA, USA). The polysaccharide was hydrolyzed by 2 M TFA at 100 °C for 12 h and used for monosaccharide composition analysis using a GC method [39].

The molecular weight of PLAP was determined by size exclusion chromatography using multiple detectors [29]: an 18-angle laser light scattering detector (GaAs3 semiconductor laser, λ = 658 nm, eighteen angles, Dawn Heleos II, Wyatt Technology Corporation, Santa Barbara, CA, USA), a differential pressure viscometer (DP) (Visco Star II, Wyatt Technology Corporation), and a refractive index detector (RI) (OPTILAB T-rEX, Wyatt Technology Corporation). The OHpak SB-G guard column (50 mm × 6.0 mm I.D., 10 μm), OHpak SB-804 HQ column (300 mm × 8.0 mm I.D., 10 μm), and OHpak SB-806 HQ column (300 mm × 8.0 mm I.D., 13 μm), all of which were from Shodex Denko Inc. (New York, NY, USA) were used in series. The mobile phase was composed of 0.1 M NaNO_3_ and 0.02% (*w*/*w*) NaN_3_ at the flow rate of 0.60 mL/min. PLAP was dissolved by the mobile phase at a concentration of 0.5 mg/mL. A refractive index increment (dn/dc) of 0.146 was used for the calculation.

### 4.4. Methylation Analysis

Methylation analysis of PLAP was conducted according to the method of previous reports [40,41] with some modifications. The partially methylated alditol acetates (PMAA) of PLAP were taken for linkage analysis using a 7890-7000A GC-MS system (Agilent, Santa Clara, CA, USA) equipped with a SP-2330 column (Supelco, Bellefonte, PA, USA; 30 m × 0.25 mm, 0.2 μm film thickness). Individual peaks of the PMAA and fragmentation patterns were identified by their mass spectra and relative retention times in the GC traces.

### 4.5. Rheological Measurements

PLAP was prepared by dissolving in ultra-pure water (1.0%, *w*/*v*) and heating at 55 °C with constant stirring, then cooled down to room temperature before steady and dynamic rheological analysis. These were also carried out with PLAP at concentrations of 0.1, 0.25, 0.5, 2.0 and 3.0% (*w*/*v*) in ultra-pure water, and at 1.0% in 0.001, 0.01, 0.1, 1.0 M NaCl or CaCl_2_ solutions. Rheological properties of the gels were measured under low-amplitude oscillatory shear using cone-and-plate (50 mm diameter with a gap of 0.046 mm, for the measurements of polysaccharide solutions in lower viscosity) or parallel plate (50 mm diameter with a gap of 0.50 mm, the measurements of polysaccharide solutions in higher viscosity or gel) geometry on an ARES Rheometer (TA Instruments, New Castle, DE, USA). All sample measurements were performed in linear viscoelastic region. The oscillatory rheological parameters used to compare the viscoelastic were: storage modulus (G′) and loss modulus (G″). Samples were loaded onto the plate at 5 °C then heated to high temperature for temperature ramp test, and temperature was controlled by a SR5 Peltier Circulator at 2 °C/min. The parallel plate (50 mm diameter) was set with a gap of 1.0 mm for the measurements.

## 5. Conclusions

PLAP is an acidic arabinoxylan, composed of 1,2,4-linked Xyl*p* and 1,3,4-linked Xyl*p* residues. PLAP showed pseudoplastic behavior and weak gelling properties. Sodium and especially calcium ions played a significant role in increasing the apparent viscosity and gel strength.

## Figures and Tables

**Table 1:** Physicochemical properties of PLAP.

Sugar (%)	Protein (%)	Uronic Acid (%)	(η) (dL/g) ^a^	Molecular Weight, *M*_w_ (×10^−6^) ^b^	Monosaccharide Composition (Molar Ratio)				
					Rha	Ara	Xyl	Glc	Gal
82.84	0.68	20.50	5.81	3.80	1.00	15.41	63.95	1.29	2.58

^a^ (η): Intrinsic viscosity; ^b^ *M*_w_: the sample recovery was 13.9%.

**Table 2:** Glycosyl-linkage compositions of PLAP.

Residue Linkage	Molar Ratio ^a^	*m*/*z*
T-linked Ara*f*	5.06	43, 71, 87, 101, 102, 118, 129, 161
1,3-linked Ara*f*	10.22	43, 69, 87, 99, 113, 118, 129, 233
T-linked Xyl*p*	10.88	43, 59, 87, 88, 101, 102, 117, 118, 161, 162
1,3-linked Xyl*p*	11.39	43, 59, 87, 101, 117, 118, 129, 173, 174
1,4-linked Xyl*p*	6.19	43, 87, 99, 102, 118, 129, 162, 189
1,2,4-linked Xyl*p*	13.30	43, 57, 71, 87, 88, 129, 130, 189, 190
1,3,4-linked Xyl*p*	38.98	43, 85, 87, 99, 118, 201, 261
T-linked Gal*p*	1.12	43, 59, 71, 87, 101, 102, 118, 129, 145, 161, 162, 205
1,4-linked Gal*p*	1.19	43, 87, 99, 102, 113, 118, 129, 162, 173, 233
1,2-linked Rha*p*	0.65	43, 88, 89, 100, 101, 115, 130, 131, 161, 190
1,3-linked Glc*p*	0.40	43, 71, 87, 101, 118, 129, 161, 234

^a^ Calculated a percentage of partially-methylated alditol acetates present, based on the peak area.

**Table 3:** Parameters of the Cross model for the range of concentrations studied.

Concentration (wt %)	η_0_^a^ (Pa.s)	α ^b^ (s)	m ^c^
0.10	0.0104	0.0174	0.3402
0.25	0.0160	0.0056	0.4732
0.50	0.0448	0.0204	0.4615
1.0	1.8271	1.3301	0.5003
2.0	44.0297	4.4526	0.8284
3.0	300.2550	9.4121	0.8788

^a^ η_0_: Zero shear rate viscosity; ^b^ α: a time constant of the power law; ^c^ m: Dimensionless constant.

**Table 4:** Parameters of the Cross model for the range of temperatures studied.

Temperature	η_0_^a^ (Pa.s)	α ^b^ (s)	m ^c^
10	7.8691	34.1772	0.4173
20	2.4583	15.1757	0.4755
30	0.5766	3.3620	0.5506
40	0.0966	0.0917	0.5945
50	0.0392	0.0094	0.5508
60	0.0226	0.0015	0.4250

^a^ η_0_: Zero shear rate viscosity; ^b^ α: a time constant; ^c^ m: Dimensionless constant.
